# PIASγ Is Required for Faithful Chromosome Segregation in Human Cells

**DOI:** 10.1371/journal.pone.0000053

**Published:** 2006-12-20

**Authors:** Laura A. Díaz-Martínez, Juan F. Giménez-Abián, Yoshiaki Azuma, Vincent Guacci, Gonzalo Giménez-Martín, Lorene M. Lanier, Duncan J. Clarke

**Affiliations:** 1 Department of Genetics, Cell Biology and Development, University of Minnesota Medical School Minneapolis, Minnesota, United States of America; 2 Proliferación Celular, Centro de Investigaciones Biológicas, Consejo Superior de Investigaciones Científicas Madrid, Spain; 3 Department of Molecular Biosciences, University of Kansas Lawrence, Kansas, United States of America; 4 Carnegie Institute, Department of Embryology Baltimore, Maryland, United States of America; 5 Department of Neuroscience, University of Minnesota Minneapolis, Minnesota, United States of America; Duke University, United States of America

## Abstract

**Background:**

The precision of the metaphase-anaphase transition ensures stable genetic inheritance. The spindle checkpoint blocks anaphase onset until the last chromosome biorients at metaphase plate, then the bonds between sister chromatids are removed and disjoined chromatids segregate to the spindle poles. But, how sister separation is triggered is not fully understood.

**Principal Findings:**

We identify PIASγ as a human E3 sumo ligase required for timely and efficient sister chromatid separation. In cells lacking PIASγ, normal metaphase plates form, but the spindle checkpoint is activated, leading to a prolonged metaphase block. Sister chromatids remain cohered even if cohesin is removed by depletion of hSgo1, because DNA catenations persist at centromeres. PIASγ-depleted cells cannot properly localize Topoisomerase II at centromeres or in the cores of mitotic chromosomes, providing a functional link between PIASγ and Topoisomerase II.

**Conclusions:**

PIASγ directs Topoisomerase II to specific chromosome regions that require efficient removal of DNA catenations prior to anaphase. The lack of this activity activates the spindle checkpoint, protecting cells from non-disjunction. Because DNA catenations persist without PIASγ in the absence of cohesin, removal of catenations and cohesin rings must be regulated in parallel.

## Introduction

Cohesion between sister chromatids must be maintained from the time of its establishment, coupled to DNA replication, until it is rapidly removed in early anaphase allowing the sister chromatids to disjoin and chromosomes to segregate to each daughter cell. It had been proposed that cohesion depends on the catenations that form between sister DNA duplexes as a consequence of their replication [1], but pivotal studies later demonstrated that proteolysis is required for chromatid separation, indicating that a protein “glue” physically links the chromatids of each chromosome [2], [3]. Mutants of genetically amenable lower eukaryotes provided support for this model [4]. An inhibitor of anaphase, Pds1, was identified in budding yeast [5], [6] and this unstable protein was found to be a substrate of a ubiquitin ligase that covalently marks proteins for proteasomal degradation [7]. Although Pds1 itself does not bind to DNA, it was shown to be an important regulator of a protease (Esp1) that cleaves the Rad21/Mcd1 component of the so-called cohesin complex that glues the sister chromatids together (reviewed in [8]). The ubiquitin ligase, now known as the Anaphase Promoting Complex/Cyclosome (APC/C), was purified from clam oocytes [9] and characterized in organisms including yeasts and frogs [10], [11].

In keeping with the model that the metaphase-anaphase transition is triggered by proteolysis, yeasts deficient in APC/C activity arrest in metaphase with bioriented chromosomes aligned correctly at the spindle equator but unable to separate their sister chromatids [12]. In mammals, efficient sister chromatid separation also requires the APC/C [13], [14] but it is likely that the control of anaphase initiation is more complex in higher eukaryotes because additional mechanisms are required to enhance the fidelity of segregation of very large genomes. Indeed, studies in the *Xenopus* egg extract system implicated an additional factor, other than the APC/C, in the regulation of chromatid disjunction. Inactivation of PIASγ in *Xenopus* egg extracts interfered with chromatid disjunction [15], [16], and this E3 sumo ligase was shown to both sumoylate Topoisomerase II and have substrates at the centromeres of mitotic chromosomes [15], [16]. Since Topoisomerase II is the only enzyme capable of removing catenations from between sister chromatids, this provided a possible link between decatenation and chromatid separation. Orthologs of PIASγ in yeasts, however, sumoylate cohesin components and other known regulators of sister cohesion, such as Pds5 [17]–[19], in addition to topoisomerase II [20], [21]. It therefore remains unknown what are the key substrates of PIASγ important for mitosis in *Xenopus* and yeast. Moreover, no mitotic functions have been ascribed to mammalian sumo ligases and PIASγ null mice have been reported to be viable [22].

Here we demonstrate that human PIASγ is required for timely anaphase onset and efficient sister chromatid disjunction. Perhaps due to a failure to release centromere cohesion in PIASγ-depleted cells, an Aurora B- and Mad2-dependent checkpoint is activated. This leads to a prolonged block in metaphase during which in some cells several chromosomes then depart from the equatorial metaphase plate but remain cohered at their centromeres. When anaphase proceeds upon chemical inhibition of Aurora B, sister chromatid separation is rarely complete, indicating a defect in loss of cohesion without PIASγ. We show that cohesin can be removed from chromosomes without PIASγ, but DNA catenations remain and can provide a cohesin-independent physical sister centromere association that is cytologically indistinguishable from that in normal chromosomes. Finally, we observe that PIASγ-depleted cells are unable to properly localize Topoisomerase II to centromere regions and mitotic chromosome cores, providing the first functional connection between PIASγ and Topoisomerase II in mammals. Inefficient removal of DNA catenations in PIASγ-depleted cells is therefore a likely cause of checkpoint activation and the defect in loss of cohesion.

## Results

### PIASγ is required for timely anaphase onset in human cells

**Figure 1:**
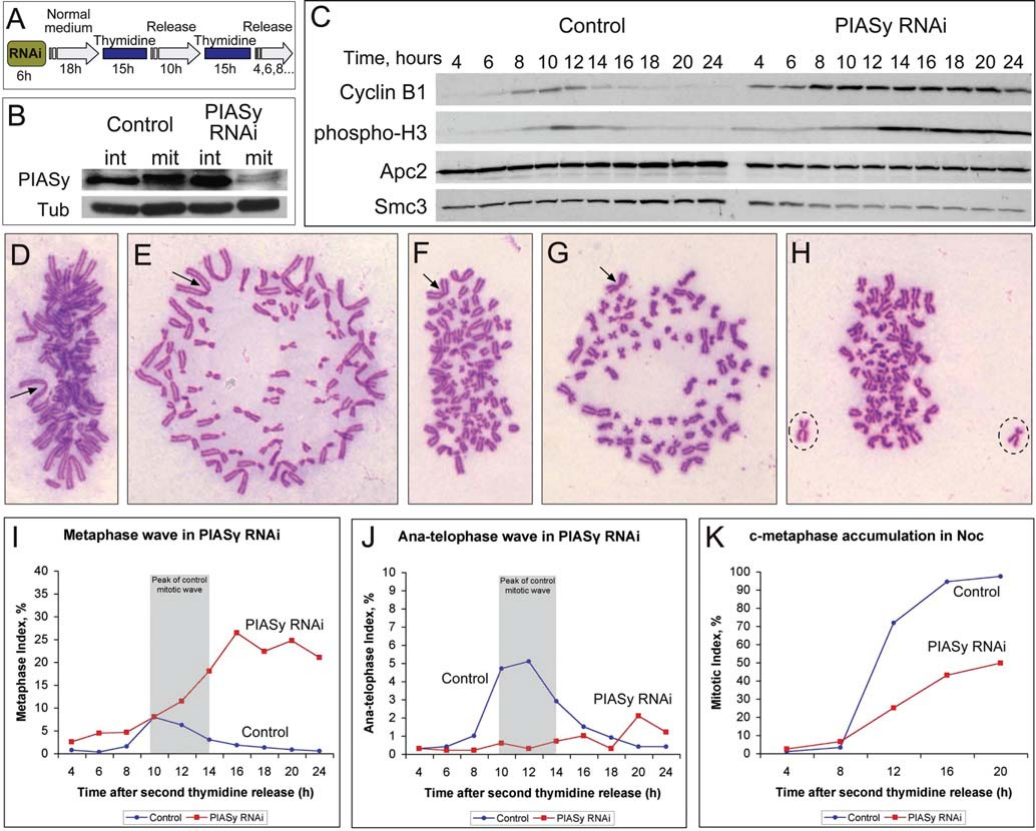
Prolonged metaphase arrest and proper chromosome alignment in PIASγ-depleted cells. Synchronous time-course experiment after release from early S-phase arrest in PIASγ-depleted HeLa cells (RNA-interference). (*A,B*) Protocol for depletion of PIASγ and Western blot analysis (mit = mitotic shake-off; int = remaining interphase cells; Tub = alpha-tubulin). (*C*) Cyclin B and Phospho-H3 transiently peaked as control-treated cells passed through mitosis, while they accumulated in PIASγ-B treated cells (these blots are from mit + int cells). Apc2 and Smc3 are loading controls. (*D–G*) Most PIASγ-depleted cells appeared to arrest in metaphase until the end of the experiment (∼12 hours after reaching mitosis). Accordingly, chromosomes became progressively over-condensed (from *D* to *G*); arrow indicates the largest metacentric chromosome in each spread. Unlike cells arrested in c-mitosis with nocodazole, PIASγ-depleted cells did not open chromosome arms. Proper chromosome alignment is evident in the side views of metaphase plates (*D,F*) and in the polar views (*E,G*). In some cells one or two chromosomes lay off the metaphase plate (*H*); since these cells usually displayed overcondensed chromosomes and because this stage followed formation of a complete metaphase plate (see time-lapse material), we describe these as de-congressed metaphases. For a detailed cytological comparison of PIASγ and control-treated cells, see Figure S1. (*I–K*) Cells progressively accumulated in metaphase (*I*) after PIASγ depletion (1000 cells scored per time-point). Shaded areas in *I,J* show control mitotic wave. (*J*) Anaphase/telophase index – Most PIASγ-depleted cells failed to initiate anaphase. (*K*) Accumulation in c-mitosis in the presence of nocodazole after control treatment or PIASγ-depletion and cell cycle synchrony in early S phase. Some PIASγ-depleted cells failed to reach mitosis as indicated by the accumulation of only ∼50% c-mitotics after 16 hours compared with over 90% in control-treated samples, indicating that PIASγ might also have roles in interphase (see Fig. S2).

Based on the hypothesis that the E3 sumo ligase PIASγ contributes to the regulation of sister separation in human cells, we sought to test if depletion of PIASγ perturbs mitotic progression. We devised a protocol that allowed depletion of PIASγ from HeLa cells simultaneously with cell cycle synchrony in early S-phase (Fig. 1*A,B*). Briefly, control or PIASγ-specific siRNA was added to asynchronously growing HeLa cells at 30% confluence, the siRNA was removed after 6 hours, then after 18 hours a double-thymidine synchrony procedure was employed. Samples were taken every 2 hours after release and processed for cytological and biochemical analysis. Upon release from early S-phase, control-treated and PIASγ-specific siRNA-treated cells first entered mitosis after ∼10 hours (Fig. 1*C,I*). While most control cells had reached the subsequent G1 phase by 14 hours, PIASγ siRNA-treated cells accumulated in mitosis (∼35%) and the mitotic index remained high up to 24 hours after the release of the thymidine block (Fig. 1*I,J* and data not shown). This mitotic delay was also evident in the high levels of cyclin B and phosphorylated histone H3 (PH3) during this time frame (Fig. 1*C*). The cells in mitosis lacked PIASγ protein as judged by Western blot, whereas PIASγ protein was detectable in the population of interphase cells (Fig. 1*B*), indicating that those cells which accumulated in mitosis were the same cells in which PIASγ protein had been efficiently depleted. Analysis of the cytology of the mitotic cells revealed that most had metaphase chromosome arrangements indistinguishable from control metaphases (Fig. 1*D,E*). That is, the chromosomes were organized into a metaphase plate arrangement and centromeric regions of the chromosomes possessed distinct primary constrictions. Many of these cells must have endured a prolonged delay in mitosis because at the later time points (18–24 hrs) they displayed over-condensed chromosomes typical of cells in which anaphase onset had been blocked (Fig. 1*F,G*). Also at later time points, similar cells with overcondensed chromosomes had several chromosomes positioned off the metaphase plate (Fig. 1*H*). Very few cells lacking PIASγ were observed with separated sister chromatids (Fig. 1*J*). (For a more detailed description of these phenotypes of PIASγ-depleted cells see Fig. S1 and S2.) These data indicate that PIASγ is needed for the metaphase-to-anaphase transition in human cells and therefore suggest that sumoylation of mitotic substrates is a key component of anaphase initiation.

### Metaphase plates form normally without PIASγ but chromosomes can then leave the plate

**Figure 2:**
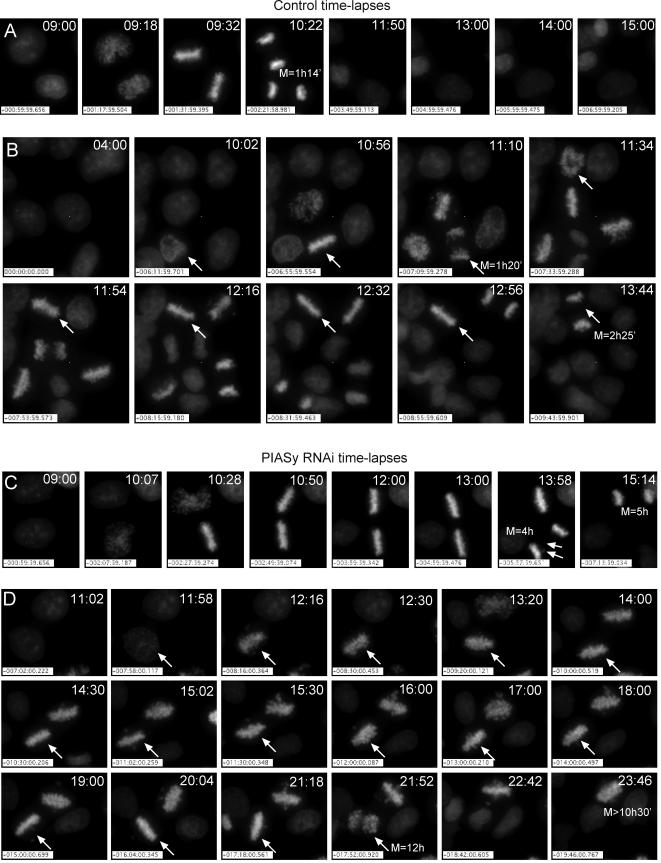
Live-cell time-lapse analysis of PIASγ-depleted cells. Selected frames of cells extracted from two fields of control and two fields of PIASγ-depleted cells that were filmed over a 16 (*A,C*) or 20 hour (*B,D*) period after release from double thymidine synchrony. (Supporting Information includes movies of the entire fields from which series *B* and *D* were taken and also the movies corresponding to *A* and *C*, and a detailed analysis of the cell cycle progression of each cell in Fig. S3 and Table S1.) (*A,B*) Control, (*C,D*) PIASγ-depleted cells. Numbers in the right top corner indicate time after the release of the second thymidine block. (*A*) Two control cells dividing. Length of mitosis (M) was 1 hour 14 minutes in both cases (Movie S1). (*B*) Six control cells dividing. The two cells with longer mitoses are indicated by arrows and the total mitotic length is indicated; cells are selected from a field shown in Movie S2. (*C*) Two PIASγ-depleted cells entering mitosis (10 hours 07 minutes and 10 hours 28 minutes, respectively). They reached metaphase 18 minutes and 15 minutes after nuclear envelope breakdown, and remained in metaphase for 3 hours 26 minutes and 4 hours 18 minutes before undergoing anaphase. The cell at the bottom displayed some lagging chromosomes (arrows) during anaphase that finally incorporated into one of the daughter nuclei. These cells are shown in Movie S3. (*D*) PIASγ-depleted cells dividing. The cell at the bottom reached metaphase, delayed in metaphase, several chromosomes departed from the plate (de-congressed metaphase), recovered a complete metaphase plate, then returned to de-congressed metaphase and finally initiated anaphase just after re-achieving metaphase alignment once more. The cell at the top spent most of the time in de-congressed metaphase after having spent more than one hour in metaphase. These cells are selected from a wider field shown in Movie S4.

That a maximum of 35% of the cells accumulated in mitosis after PIASγ depletion (Fig. 1*I* and data not shown) suggested either that the cells entered mitosis more slowly than in controls, or that the cells eventually exited mitosis after delaying in metaphase (or a combination of both of these factors). Slower entry into mitosis was consistent with the result that, when the above time course experiment was repeated in the presence of nocodazole, PIASγ-depleted cells accumulated in mitosis to a lesser extent than controls (Fig. 1*K*). However, to determine if some cells exited mitosis after PIASγ-depletion, and to gain a more detailed understanding of the mitotic defect in live cells, we performed 16–20 hour time-lapse analyses of HeLa cells possessing a fluorescent tagged histone (H2B-GFP) [23] following early S-phase synchrony (Fig. 2, Fig. S3, Table S1, and Movies S1–S4).

In controls, mitosis proceeded normally (Fig. 2*A,B*, Fig. S3 and Table S1), and cell synchrony was evidently sharp since 33/51 (64%) of the cells within a particular field progressed into mitosis during a short 4 hour time period (10–14 hours after release from early S-phase). PIASγ-depleted cells also reached mitosis quite synchronously with over half of the cells in a single field initiating mitosis within a 5 hour time frame after release from early S-phase (Fig. S3). Consistent with the time course experiment described in Fig. 1, most PIASγ-depleted cells became strongly delayed in mitosis. The average time spent in mitosis was 6 hours 35 minutes (s.d. = 3 hours 56 minutes; n = 26), compared with 69 minutes in control cells (s.d. = 24 minutes; n = 36).

PIASγ-depleted cells usually performed prometaphase with normal timing forming bona fide metaphase plates (Fig. 2*C,D*, Fig. S3 and Table S1). In these cells, metaphase lasted 1–6× longer than in control cells (on average 110 minutes with s.d. = 78 minutes, compared with 44 minutes with s.d. = 25 minutes in controls). The maximum length of metaphase recorded was 4 hours 10 minutes. Following the prolonged metaphase period, two alternative outcomes were observed, either (i) the cell initiated anaphase (Fig. 2*C*) and occasionally, lagging chromosomes were observed (see bottom cell in Movie S3 that corresponds to Fig. 2*C*; see arrows) or anaphase initiation was asynchronous (see Fig. S1*P*), or (ii) several chromosomes departed from the metaphase plate and migrated toward a spindle pole (Fig. 2*D*, top cell). In this latter category, the cells appeared to have reverted to a prometaphase-like state; in some cases these cells recovered complete metaphase alignment of all chromosomes and at a later stage, after further delay in metaphase, were able to perform anaphase (Fig. 2*D*, bottom cell). Other cells in this category remained in this “de-congressed” metaphase state until the end of the time-lapse movie (Fig. 2*D*, top cell).

Together with the cytological analysis in Figure 1 and Figure S1, these live cell imaging studies provide a detailed description of the mitotic defects caused by PIASγ-depletion. Most cells reach metaphase in a timely manner and form apparently normal metaphase plates. After a prolonged metaphase delay cells either performed anaphase or a small number of chromosomes left the plate and migrated to the spindle poles. This de-congressed metaphase state was maintained in most cells for many hours.

### The spindle checkpoint ought to be satisfied without PIASγ but centromeres remain cohered

**Figure 3:**
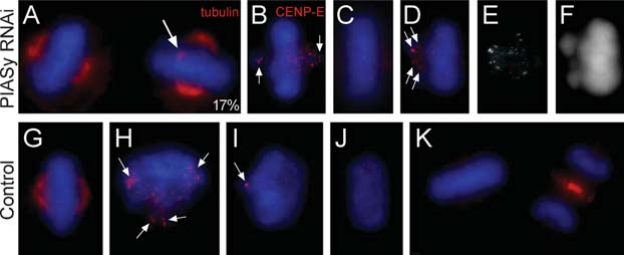
Normal spindle morphologies and reduced CENP-E staining at the kinetochores of aligned chromosomes in PIASγ-depleted cells. (*A,G*) Mitotic spindles (stained with anti-alpha-tubulin antibody) were indistinguishable in control and PIASγ-depleted metaphase cells, except that a small % at late time-points possessed extra poles (arrow in *A*): (*A,G*) Red = alpha-tubulin, Blue = DAPI. (*B–F,H–K*) CENP-E staining (Red) is detected strongly on kinetochores of chromosomes away from the metaphase plate in both control-treated and PIASγ-depleted cells (*B,H,I*), but was much reduced in chromosomes at the plate (*C,J,K*). After prolonged arrest in metaphase some of PIASγ-depleted cells had one or two chromosomes that left the plate and regained strong CENP-E staining at their kinetochores (*D–F* and see Fig. S1*N,O*). The same cell is shown in *D* (merged image), *E* (CENP-E straining) and *F* (DAPI straining).

That metaphase plates formed normally without PIASγ but anaphase failed to initiate on time seemed counterintuitive. The time-lapse studies indicated that when initiated, anaphase proceeded normally in some PIASγ-depleted cells, indicating a functional mitotic spindle and motors. In agreement, immuno-staining of alpha-tubulin revealed mitotic spindles that were indistinguishable from control metaphases in cells lacking PIASγ (Fig. 3*A,G*). Moreover, the kinetochores of chromosomes at the metaphase plate in both control cells and cells that lacked PIASγ clearly possessed far less CENP-E than kinetochores of chromosomes away from the plate (Fig. 3*B–F,H–K*). This staining pattern is typical of spindle checkpoint inactivation after congression of each chromosome to the metaphase plate [24], [25]. That metaphase plates formed in PIASγ-depleted cells which were indistinguishable from control metaphase plates, also argues that the spindle checkpoint ought to have been silenced because biorientation of chromosomes at the metaphase plate can only occur if proper kinetochore-microtubule attachments are made, resulting in a balance of opposite forces exerted by the spindle on each cohered sister and producing the proper spindle tension on each chromosome. Therefore, the prolonged metaphase delays in PIASγ-depleted cells indicate that a mitotic checkpoint is able to act in metaphase: this has only been previously reported to occur in response to inhibition of Topoisomerase II [26]–[28].

### An Aurora B-dependent and Mad2-dependent checkpoint is activated following PIASγ depletion

**Figure 4:**
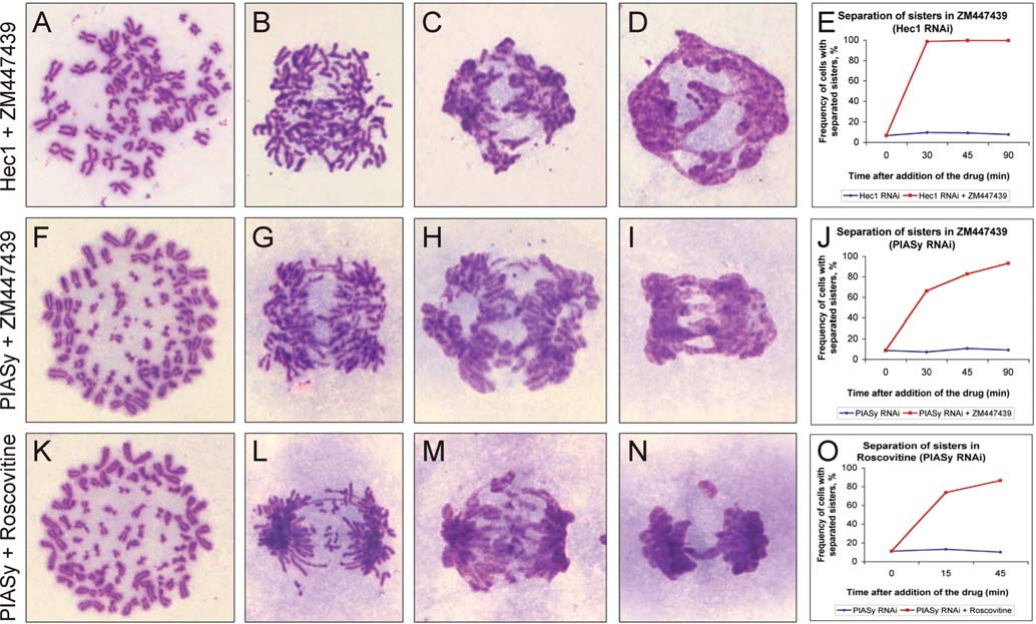
Anaphase is induced by Aurora B or Cdk inhibition in PIASγ-depleted metaphase cells. (*A–E*) Depletion of Hec1 using RNAi [30] induces a persistent spindle checkpoint arrest in prometaphase (*A*). The Aurora B inhibitor ZM447439 (5.6 µM) [29] bypassed this arrest: cells performed anaphase (*B,C*) and exited mitosis within 30 minutes (*D,E*). (*F–J*) Cells arrested in metaphase by PIASγ depletion similarly perform anaphase and exit mitosis upon ZM447439 addition, but with slightly slower kinetics than Hec1-depleted cells, demonstrating that: (1) spindle-kinetochore interactions are functional after PIASγ-depletion, (2) sister chromatids are able to migrate to opposite spindle poles, (3) the metaphase arrest after PIASγ-depletion is due to a checkpoint response. (*K–O*) Anaphase and mitotic exit are induced by the Cdk inhibitor roscovitine (150 µM) in PIASγ-depleted metaphase arrested cells.

The above findings indicated that a lack of PIASγ might have triggered a mitotic checkpoint. Although the spindle checkpoint appeared to have been silenced in metaphases lacking PIASγ, based on the reduction of CENP-E staining at kinetochores, we found that co-depletion, using RNAi, of Mad2 and PIASγ abrogated the accumulation of cells in mitosis following release from a double thymidine synchrony (data not shown). We also found that the small molecule inhibitor of Aurora B, ZM447439 [29], was able to relieve the metaphase arrest (Fig. 4). In control cells that were depleted of Hec1, a treatment that induces a persistent spindle checkpoint arrest [30], addition of ZM447439 resulted in rapid sister separation followed by mitotic exit in almost all cells (Fig. 4*A–E*). Similarly, after we collected PIASγ-depleted mitotic cells by mitotic shake-off then added ZM447439, most cells rapidly performed sister chromatid separation and segregation, followed by mitotic exit (Fig. 4*F–J*). Anaphase and mitotic exit could have been a result of cyclin B degradation upon ZM447439 treatment. Consistent with this, addition of the Cdk inhibitor roscovitine had similar effects on PIASγ-depleted cells (Fig. 4*K–O*), inducing chromatid separation, segregation to the spindle poles and mitotic exit. These experiments therefore resolved several important issues. First, PIASγ-depleted cells are largely capable of removing the cohesion from between the sisters as long as Cdk-cyclin B is inactivated. Second, as seen in the time-lapse studies, PIASγ-depleted cells treated with ZM447439 or roscovitine must have possessed functional mitotic spindles because chromosomes segregated to the spindle poles before decondensing in telophase. Third, the metaphase delay imposed by PIASγ depletion is a checkpoint response.

Checkpoints can monitor defects in biological processes and respond by delaying cell cycle progression [8]. The metaphase delay in PIASγ-depleted cells is typical of a checkpoint response because anaphase onset is blocked despite being mechanically able to proceed, the delay can be bypassed by chemical inhibition of one of the checkpoint components (in this case Aurora B), and the delay is spontaneously overcome in some cells even though a successful anaphase could not always be completed. When overridden by ZM447439 or roscovitine, chromatid disjunction was rarely efficient (Fig. 4*F–O*). Typically, bypass of a checkpoint delay reveals the cellular aberration that activated the checkpoint. In the case of the delay in PIASγ-depleted cells, checkpoint bypass revealed multiple chromatin bridges between the dividing nuclei in almost every cell. Laggard chromosomes were also seen in some of the anaphases in the time-lapse movies of PIASγ-depleted cells that had previously delayed in metaphase for a prolonged period. These data indicate that the defect in PIASγ-depleted cells might be an inability to efficiently separate the sister chromatids.

### PIASγ is required for sister separation in hSgo1-depleted cells

**Figure 5:**
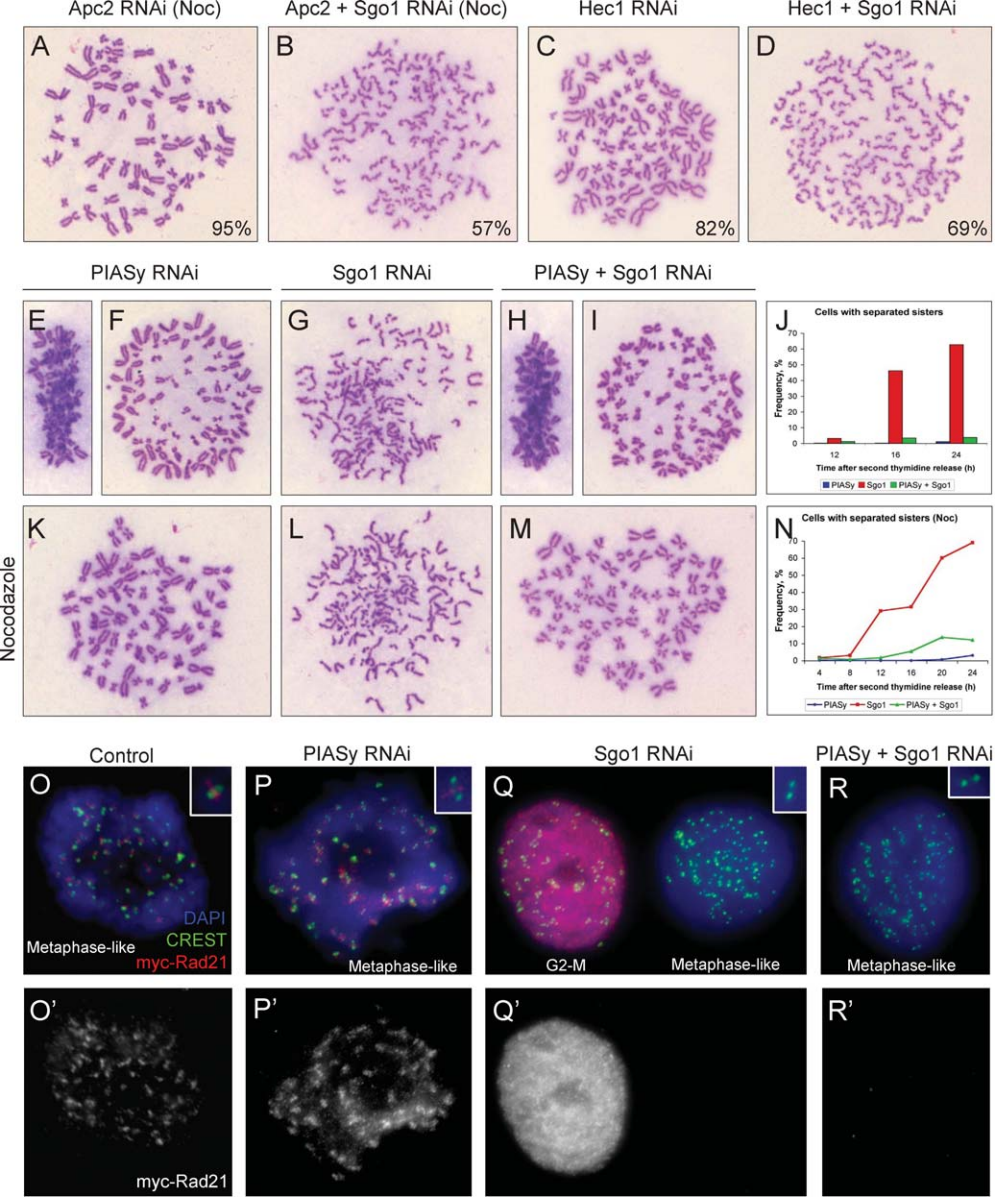
Sister chromatids cannot separate in PIASγ-depleted cells lacking the cohesin protector hSgo1. (*A,B*) HeLa cells arrest in mitosis with separated sisters when an essential component of the APC/C (Apc2) is depleted together with the cohesin protector hSgo1. RNAi was performed as previously described [13] and cells allowed to reach mitosis after early S-phase synchrony in the presence of nocodazole: (*A*) c-mitosis arrest with nocodazole after Apc2 depletion; (*B*) complete sister chromatid separation in the presence of nocodazole after hSgo1 and Apc2 co-depletion. (*C,D*) hSgo1 is required for sister cohesion when a persistent spindle checkpoint is induced by Hec1-depletion (cells were synchronized and Hec1/hSgo1 depleted as described in Figure 3 and [13]): (*C*) Prometaphase arrest after Hec1-depletion; (*D*) complete sister separation after hSgo1 and Hec1 co-depletion. Numbers on these micrographs (*A–D*) indicate % cells that arrested with these phenotypes 20 hours after release from S-phase. (*E–N*) hSgo1 depletion does not result in sister separation when PIASγ is co-depleted, either in the absence (*E–J*) or presence of nocodazole (*K–N*): (*E,F*) metaphase arrest after PIASγ-depletion; (*K*) c-mitosis arrest in nocodazole after PIASγ-depletion; (*G,L*) complete sister separation after hSgo1-depletion, with or without nocodazole; (*H,I*) metaphase arrest after hSgo1 and PIASγ co-depletion; (*M*) c-mitosis arrest in nocodazole after hSgo1 and PIASγ co-depletion. (*J*) After release from early S-phase, hSgo1-depleted cells arrest in mitosis with separated sisters, while almost all PIASγ-depleted and hSgo1/PIASγ co-depleted cells arrest with cohered sisters. Similar results were obtained in cells treated with nocodazole upon release from early S-phase (*N*). Nocodazole used at 0.25 µM. (*O–R′*) Immunostaining of myc-tagged Rad21 in HeLa cells. (*O–R*) Merge of DAPI (blue), CREST (green), myc-Rad21 (red). (*O′–R′*) myc-Rad21 staining only. (*O,O′*) Control cell. Rad21 localizes strongly between kinetochores, revealed by CREST signals, and weakly between chromosome arms. The term “metaphase-like”, as in the other panels, refers to the fact that metaphases are not unequivocally identifiable after the pre-extraction and fixation procedures required before immunostaining. (*P, P′*) PIASγ-depleted cell at metaphase displaying a pattern of myc-Rad21 localization similar to that of control cells. (*Q–Q′*) Sgo1-depleted cells. The cell on the left is most likely an early prophase cell (judging by the closely paired sister kinetochores) and shows high myc-Rad21 staining throughout the nucleus, while the cell on the right does not have any detectable myc-Rad21 (most chromosomes have paired kinetochores while several have already separated, typical of precocious sister separation in prometaphase after hSgo1 depletion). (*R,R′*) Metaphase cell after PIASγ and hSgo1 co-depletion. No detectable myc-Rad21 staining is observed though sister centromere cohesion is maintained (kinetochores are paired).

The lack of PIASγ resulted in checkpoint activation at the metaphase-to-anaphase transition and the cells failed to remove cohesion from the centromeres. However, the above experiments did not address directly if the lack of PIASγ perturbed the removal of cohesin. A number of important studies have shown that sister chromatid cohesion cannot be maintained in cells depleted of the cohesin protector hSgo1, even under conditions in which the spindle checkpoint is active [13], [31]–[35]. These results demonstrated that without hSgo1, cells are oblivious to spindle checkpoint controls and that cohesin is rapidly removed from chromosomes upon nuclear envelope breakdown. If PIASγ depletion arrested cells in metaphase by activating the spindle checkpoint, but has no direct role related to chromosome cohesion, then depletion of hSgo1 should relieve sister chromatid cohesion after PIASγ knock-down. As previously described, we could efficiently deplete HeLa cells of hSgo1 by RNA interference (data not shown) and found that, similar to previous reports, the cells accumulated in mitosis with all of their sister chromatids fully separated, even in the presence of nocodazole [13]. In the absence of hSgo1, sister chromatids were similarly able to separate in cells in which APC/C activity had been eliminated by depletion of the Apc2 component of the catalytic site and by the simultaneous addition of nocodazole (Fig. 5*A,B*). Furthermore, if the spindle checkpoint was persistently activated by depletion of Hec1 (Fig. 5*C*), simultaneous depletion of hSgo1 resulted in complete loss of sister cohesion (Fig. 5*D*). Each of these experiments confirms that the known spindle checkpoint pathways are unable to preserve cohesion in the absence of cohesin guardian hSgo1.

We depleted both PIASγ and hSgo1 in synchronized HeLa cells using RNAi (data not shown) and a double thymidine block protocol, then released into the cell cycle either with or without nocodazole. As previously reported, hSgo1-depleted cells accumulated in mitosis with separated sister chromatids, whether or not nocodazole was present in the medium, and cell cycle progression was blocked in a telophase-like state (Fig. *5G,J,L,N*). Unexpectedly, however, the doubly depleted HeLa cells lacking PIASγ and hSgo1 accumulated in metaphase/de-congressed metaphase with cohered sister chromatids (Fig. 5*H–J,M,N*), just like the cells depleted of PIASγ only (Fig. *5E,F,K*). Thus, remarkably, even in the absence of the cohesin protector hSgo1, PIASγ is required for sister chromatid separation. The same result was observed when PIASγ was depleted simultaneously with Sororin, a protein that interacts physically with the cohesin complex and is required for sister chromatid cohesion in mitosis (data not shown) [36], [37]. Together these experiments indicate that PIASγ might be directly involved in the removal of cohesion.

### PIASγ is not required for removal of cohesin from centromeres

The lack of sister separation in PIASγ/hSgo1 doubly depleted cells could be explained in one of two ways: either, (1) PIASγ is required for cohesin removal even in the absence of the cohesin guardian, or (2) sister chromatids remain cohered at the centromeres in the absence of cohesin. To test this we immuno-localized Rad21 in cells after PIASγ-depletion, hSgo1-depletion, or in doubly depleted cells. As expected, mitotic chromosomes in hSgo1-depleted cells lacked any detectable cohesin except before breakdown of the nuclear envelope, in which case cohesin was strongly detected throughout the nucleus (Fig. 5Q,Q′). PIASγ-depleted mitotic cells, however, like control cells, possessed clearly defined regions of centromeric Rad21 between the paired kinetochores of each cohered chromosome (Fig. 5*P,P′*). Some Rad21 was also seen between the chromosome arms (Fig. 5*O,O′,P,P′*). Strikingly, Rad21 could not be observed between the paired kinetochores or the arms of the cohered sisters in hSgo1/PIASγ doubly depleted cells (Fig. 5*R,R′*). Therefore, PIASγ is not required for removal of cohesin from chromosomes that occurs in the absence of hSgo1, but PIASγ is required for sister chromatid separation under the same experimental conditions. Thus, cohesion between sister kinetochores was maintained in the absence of detectable Rad21.

### DNA catenations might maintain the centromeric primary constriction and cohesion at the centromere in the absence of cohesin

**Figure 6:**
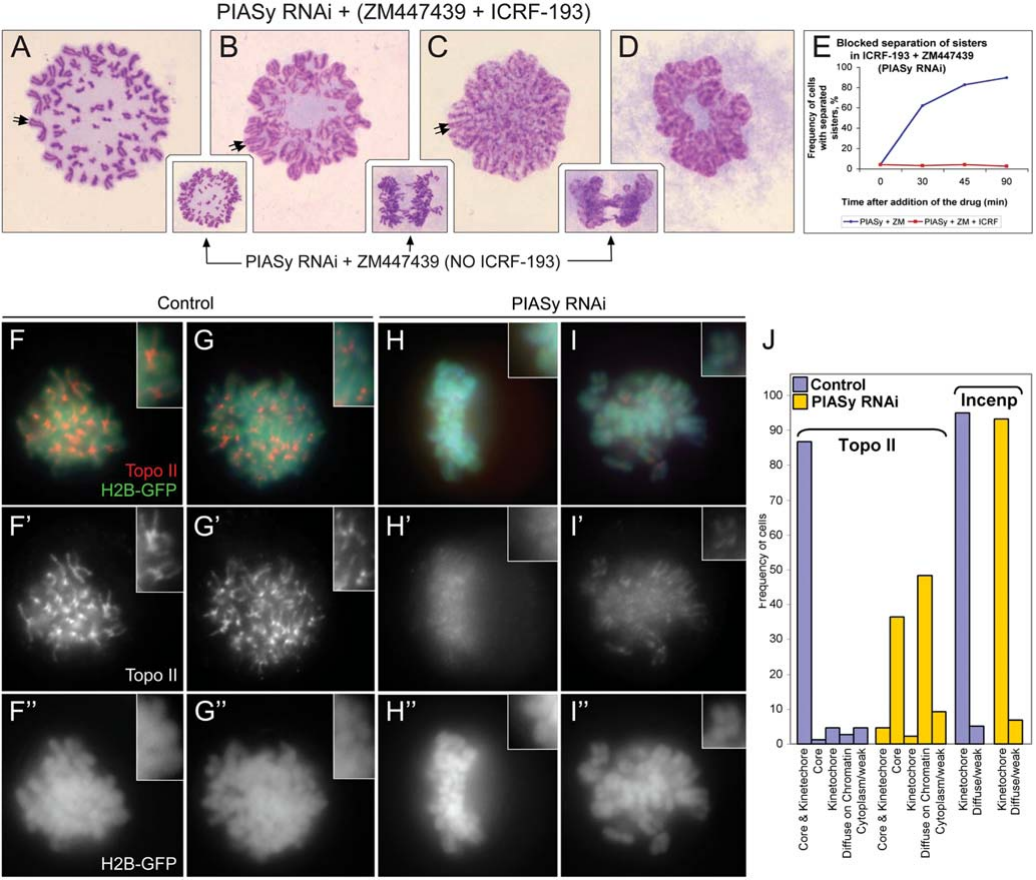
Sister chromatids cannot separate in PIASγ-depleted cells lacking Topoisomerase II activity. Metaphase arrested cells depleted of PIASγ were collected as described in Figure 4, then the Aurora B inhibitor ZM447439 and the Topoisomerase II inhibitor ICRF-193 were added and samples taken for cytological analysis. (*A*) metaphase; (*B,C,D*) failed sister disjunction during anaphase and subsequent exit from mitosis. In *B* and *C* sister chromatids can still be observed to be cohered while the chromatin is largely decondensing (double arrows). For comparison, small inserted panels show metaphase (left-most panel) and anaphase (two panels on the right) in the presence of ZM447439 alone. (*E*) Frequency of cells in which sisters were able to disjoin upon addition of ZM447439, with or without ICRF-193. (*F–I*) Immunostaining of Topoisomerase IIα in HeLa cells expressing H2B-GFP (inserts show a slight enlargement of a selected region from the same micrograph). (*F–I*) Merge - Topoisomerase IIα (red) and H2B-GFP (green). (*F′–I′*) Topoisomerase IIα staining only. (*F′′–I′′*) H2B-GFP only. (*F–G*) Control mitotic cells showing Topoisomerase IIα localized to cores and concentrated at centromere regions; (*H*) PIASγ-depleted mitotic cell with diffusely localized Topoisomerase IIα on the chromatin; (*I*) PIASγ-depleted mitotic cell with Topoisomerase IIα localized to cores but not strongly enriched at centromere regions. (*J*) Classification of Topoisomerase IIα and INCENP immunostaining patterns in control and PIASγ-depleted mitotic cells. The “Kinetochore” and “Core & Kinetochore” categories required that Topoisomerase IIα was strongly concentrated at the centromere regions.

Since PIASγ was required for sister separation under two different conditions (absence of Sororin or hSgo1) in which cohesin-based cohesion cannot hold sisters together, and because we were unable to detect cohesin Rad21 at centromeres in PIASγ/hSgo1 depleted cells, we speculated that cohesin was not the sole component providing sister cohesion after PIASγ depletion. In yeast, components and regulators of the cohesin complex are modified by sumo ligases and, in addition, yeast Topoisomerase II is sumoylated. A known mechanism that joins sister chromatids, though not known to be strictly regulated, is DNA catenation, that arises as sister DNA molecules are synthesized during S-phase. In budding yeast and *Xenopus,* PIASγ-mediated sumoylation of DNA Topoisomerase II, the only enzyme capable of removing catenations from between sister chromatids, is thought to target Topoisomerase II to centromeres or pericentric regions of chromosomes during mitosis [16], [21]. It was therefore plausible that catenations, in addition to cohesin, linked the sister chromatids in PIASγ-depleted cells. This could explain why PIASγ and hSgo1 doubly depleted cells retained sister chromatid cohesion in the absence of cohesin and would be indicative of a need for PIASγ for catenation removal. To test this hypothesis we employed a specific inhibitor of Topoisomerase II, ICRF-193, that locks the enzyme in the so-called “closed-clamp” form, preventing concatenated sister duplexes from being resolved. We depleted PIASγ from HeLa cells before a double thymidine synchrony and then collected the cells that became arrested in mitosis after release from the S-phase block. As described in Figure 4, the Cdk inhibitor roscovitine or the Aurora B inhibitor ZM447439 caused such mitotic cells to separate most of their sister chromatids and then segregate them to the spindle poles, demonstrating that sister chromatid cohesion was largely removed. If PIASγ-depleted mitotic cells possess catenations that hold the sister DNA molecules together, then inhibition of Topoisomerase II ought to block the sister separation that is forced upon roscovitine or ZM447439 treatment. We added roscovitine (data not shown) or ZM447439 to the PIASγ-depleted mitotic cells simultaneously with ICRF-193 and prepared samples for cytology. Strikingly, inhibition of Topoisomerase II completely blocked sister chromatid separation in every cell observed. That Topoisomerase II was required for sister separation under these conditions, indicates that catenations were indeed present in the PIASγ-depleted metaphase-arrested cells (Fig. 6*A–E*).

### PIASγ is required for efficient localization of Topoisomerase II to centromere regions and chromosome cores in mitotic human cells

If indeed catenations could not be removed efficiently from the centromeres of PIASγ-depleted cells, then these catenations must have persisted despite the fact that Topoisomerase II is active in mitotic cells. One mechanism that could account for this apparent paradox would be if PIASγ helps to direct the decatenatory activity of Topoisomerase II to centromeric catenations. To test this hypothesis, we immuno-localized Topoisomerase IIα in control mitotic cells and in cells depleted of PIASγ (Fig. 6*F–J*). During mitosis, Topoisomerase II is associated with the axial cores that run the length of condensed chromosome arms, but is also specifically concentrated at the centromere regions [38]–[43]. Using polyclonal antisera directed at Topoisomerase IIα, we reproducibly observed this staining pattern (core localization and intense staining at the centromere region) in almost 90% of the control cells (Fig. 6*F,G,J*). Strikingly, however, fewer than 5% of PIASγ-depleted mitotic cells had this staining pattern. Instead, almost 40% of PIASγ-depleted mitotic cells had prominent staining of the chromosome cores along the chromosome arms, but lacked the intense staining at the centromere regions (Fig. 6*I,J*). A further 48% of the PIASγ-depleted cells had a pattern of diffuse staining coincident with the chromatin, but not well localized to the cores or centromere regions (Fig. 6*H,J*). Other proteins that specifically localize to centromere regions during mitosis, such as INCENP and CENP-F, localized to centromeres equally well in control and PIASγ-depleted mitotic cells (Fig. 6*J* and data not shown). These data are consistent with a need for PIASγ for proper localization of Topoisomerase II to centromere regions of chromosomes in mitosis and further suggest that localization to chromosome cores is less efficient in the absence of PIASγ.

## Discussion

### Two different mechanisms regulate sister chromatid cohesion

Separation of sister chromatids at the metaphase-anaphase transition is the key moment of the mitotic cell cycle and its accuracy allows faithful partitioning of the duplicated genome. Groundbreaking studies have described a cohesin-based system that physically holds sister chromatids together and the mechanisms that regulate dissolution of this glue in preparation for anaphase [44]. In yeasts, firm genetic evidence has established that cohesin is the predominant, if not the sole, factor that accounts for sister cohesion and DNA catenations are removed from yeast chromosomes well before anaphase onset [45]. But in vertebrates, unlike in yeast, DNA catenations as well as cohesin complexes are present at centromeres until anaphase [46], [47]. Whether centromeric DNA catenations play an important functional role, rather than persisting as a mere coincidence, has not been understood because evidence that the catenation state of DNA is regulated through the cell cycle has been lacking. It is clear, however, that mechanisms must exist to ensure the efficient removal of centromeric catenations once the commitment to separate sister chromatids has been made. Here we provide the first hint that DNA catenations between human sister chromatids are specifically targeted for removal at or just prior to anaphase onset and that centromeric decatenation is regulated by distinct mechanisms from those which orchestrate removal of cohesin from the centromere. Our data suggest that a sumo ligase, PIASγ, promotes sister decatenation in preparation for or during sister separation, by specifically targeting Topoisomerase II to centromeric regions where catenations remain until anaphase.

**Figure 7:**
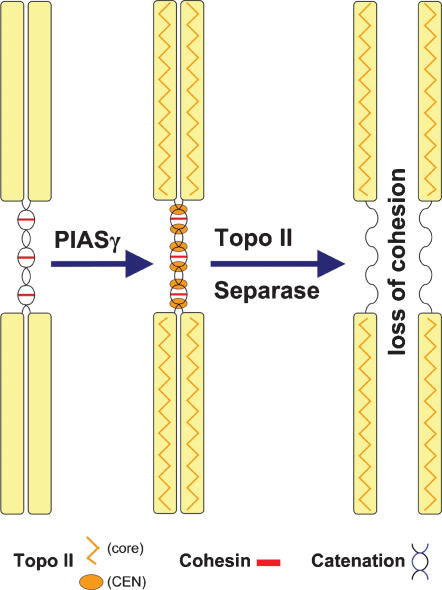
Role of PIASγ in chromosome segregation A model illustrating the role of PIASγ in mitosis and the relationships between cohesin complexes and DNA catenations. In theory, sister chromatids remain cohered when either cohesin or DNA catenations are removed. Faithful chromosome segregation therefore relies on the concerted action of separase (which removes centromeric cohesin), PIASγ (which helps localize Topo II to centromere regions) and Topo II (which resolves DNA catenations at the centromere).

In metaphase mammalian cells cohesin complexes persist at the centromeres [48]. DNA catenation must also exist at the centromeres in metaphase because Topoisomerase II inhibitors block chromatid disjunction when added to cells just prior to anaphase onset [46], [47]. We have shown that HeLa cells depleted of PIASγ suffer a prolonged metaphase block in which cohesin and catenations are maintained at the centromere, just like in normal metaphase cells. However, upon removal of cohesin from these centromeres by co-depletion of hSgo1, DNA catenations remain between the sister chromatids. Thus, although the regulation of cohesin binding at centromeres clearly depends on the cohesin guardian hSgo1, the persistence of DNA catenations at centromeres does not depend on hSgo1 (in the absence of PIASγ) nor does it depend on the presence of cohesin. In the apparent absence of cohesin at the centromere, PIASγ/hSgo1 doubly depleted cells block in metaphase: chromosomes with cohered sisters remain at the metaphase plate, and must therefore be under tension from the mitotic spindle, but remain physically tethered by catenations. These data provide two novel conclusions about the mechanism of sister cohesion. Firstly, the persistence of catenations and cohesion are regulated independently, cohesin by factors such as separase, hSgo1 and Plk1, and catenations by PIASγ and Topoisomerase II (Fig. 7). Secondly, catenations do not persist in metaphase simply because the sisters are held in close proximity by cohesin complexes.

### Does the spindle assembly checkpoint monitor the ability to decatenate centromeric DNA?

The kinetics of progression through mitosis after PIASγ depletion revealed typical features of checkpoint activation: first, cells delayed progression to the next cell cycle stage (metaphase was prolonged compared to controls); second, the delay could be overridden spontaneously (as seen in the time-lapse movies) and by chemical treatment (Aurora B inhibition, Cdk inhibition); third, overriding the checkpoint revealed defects that the checkpoint was presumably aiming to avoid (anaphase chromatin bridges seen after chemical override, and laggard chromosomes and asynchronous anaphases after spontaneous override).

Our data indicate that without PIASγ, cells become blocked in mitosis due to activation of an Aurora B- and Mad2-dependent checkpoint. Previous studies of the role of Aurora B in spindle checkpoint control indicated that Aurora B helps to sense and/or correct mono-polarly orientated chromosomes and merotelically attached kinetochores. We have not determined the precise molecular event that triggers activation of the checkpoint after PIASγ depletion, but at a cytological level based on chromosome spreads, immuno-localization of CENP-E and live cell imaging, we could not detect a mitotic defect prior to metaphase chromosome alignment. That all of the chromosomes biorient at the metaphase plate in PIASγ-depleted cells, discounts the possibility that monopolar orientation of chromosomes and, possibly, merotelic attachments are the cause of checkpoint activation. The majority of HeLa cells depleted of PIASγ reached metaphase with kinetics indistinguishable from controls cells, then blocked in metaphase for a prolonged period (on average 2.5× the normal length of metaphase). In these metaphase cells filmed by time-lapse analysis, the typical movements of the metaphase plate occurred as in control cells, presumably indicating normal spindle and motor function. Indeed, proper alignment of all the chromosomes at the metaphase plate implies correct kinetochore, spindle and motor behavior as well as proper tension on each chromosome. The only molecular defects that we could provide evidence for in PIASγ-depleted cells were the inability to remove centromeric catenations and a lack of Topoisomerase II localization to centromere regions (and mitotic chromosome cores in some cells). It is therefore possible that the anaphase checkpoint is induced in response to persistent centromeric catenations or the inability to remove these catenations efficiently. Other studies lend support to this model in that inhibition of Topoisomerase II, the only enzyme that can decatenate DNA, prior to anaphase onset induces a metaphase checkpoint delay [26]–[28].

The separation of sister chromatids that occurs during mitosis in the *Xenopus* egg extract system also depends on PIASγ. But in this in vitro system, the lack of PIASγ did not result in a prolonged metaphase block, as we observed in HeLa cells depleted of PIASγ [15]. This difference may simply reflect a limitation of the egg extract system or could, more interestingly, mean that *Xenopus* embryonic-like cell cycles lack the ability to activate a checkpoint in response to PIASγ-depletion.

### PIASγ might promote efficient decatenation at the centromere

Similar to PIASγ-depleted cells, Topo II inhibitor-treated cells block in metaphase with high levels of APC/C substrates such as securin and cyclin B [27]. An important difference between these two metaphase checkpoint arrested states, however, is that the Topo II inhibited cells are unable to separate their sister chromatids upon checkpoint bypass (due to the persistent catenations) whereas PIASγ-depleted cells can separate most of their sister chromatids upon inactivation of Aurora B or Cdk-cyclin B. It must therefore be the case that PIASγ function is not absolutely required for Topo II-mediated decatenation. We therefore suggest that PIASγ functions to promote efficient Topo II-mediated decatenation at the centromere, but that without PIASγ, decatenation can still occur but perhaps without the precision necessary for faithful chromosome segregation at the moment of anaphase onset. In agreement with this hypothesis, Topoisomerase II localized diffusely on the chromosomes of PIASγ-depleted cells rather than being concentrated at centromere regions and chromosome cores. It is noteworthy that although some of the PIASγ-depleted cells that we filmed by time lapse-microscopy could perform anaphase, we observed defects in the synchrony of sister separation; accordingly, PIASγ-depleted cells treated with Aurora B inhibitors could segregate their chromosomes, but not efficiently and chromosome bridges were always seen between the dividing cells. Together, these data indicate that PIASγ functions to promote faithful chromosome segregation, presumably by increasing the efficiency of decatenation, particularly at centromeres, but is dispensable for sister separation. It seems reasonable that human cells have evolved a mechanism to determine if decatenation cannot proceed efficiently, when Topo II activity is limited or in the absence of PIASγ, and to respond by delaying anaphase onset by activation of a checkpoint. Such a defect could be detected by monitoring the presence of catenations, the proper localization of Topoisomerase II or by assessing PIASγ activity.

In the experiments where we bypassed the prolonged metaphase delay seen in the absence of PIASγ, many of the PIASγ-depleted cells had been in metaphase for a considerable period of time before we added roscovitine or ZM447439. We can infer, then, that the catenations must have persisted despite the fact that Topoisomerase II is active in control metaphase cells. It is therefore plausible that sumoylation of Topoisomerase II helps to direct its decatenatory activity to centromeric catenations. This idea is consistent with the finding that PIASγ sumoylates Topoisomerase II in *Xenopus* and yeast. However, we have not ruled out the possibility that PIASγ promotes proper Topoisomerase II localization indirectly in human cells.

In summary, we have identified a human E3 sumo ligase required for efficient and timely anaphase separation of sister chromatids. Cells that lack PIASγ arrest in metaphase with cohered sisters even in the absence of the cohesin guardian hSgo1, and without cohesin Rad21 being detectable at centromeres. Topoisomerase II does not concentrate at centromere regions in such cells and catenations remain between the sister chromatids. Therefore, we provide the first evidence that the mechanism of loss of cohesion in human cells involves removal of DNA catenations stimulated by PIASγ. Moreover, our data indicate that catenations can act redundantly with cohesin complexes and that the failure to efficiently remove catenations triggers the spindle checkpoint.

## Materials and Methods

### Cell culture and siRNA methods

Cells were grown at 37°C with 5% CO_2_ in DMEM containing high glucose, L-glutamine, sodium pyruvate and pyridosine hydrochloride (Gibco) plus Penicillin-Streptomycin (100 U/mL–100 µg/mL, Gibco). Cell synchronies were performed by double thymidine (2 mM) arrest and release into complete medium. The siRNA and synchrony protocol used for PIASγ depletion are depicted in Figure 1. PIASγ depleted using two different siRNA sequences gave similar results. Data presented here used PIASγ-B: 5′-GCUCUACGGAAAGUACUUA (dTdT)-3′. siRNAs were transfected using Lipofectamine2000 (Invitrogen) using the manufacturer instructions. 6 hours after siRNA transfection, medium was changed to normal medium containing half the normal concentration of Penicillin-Streptomycin. Nocodazole was used at 0.5 µM in DMSO.

### Cytology

For cytological analysis cells were fixed with Carnoy's and chromosome spreads prepared or with paraformaldehyde for immuno-staining, as previously described [13]. Antibodies: anti-CENP-E, 1∶250 dilution (Immuquest); anti-myc, 1∶1000 dilution (Gramsch Laboratories); anti-Topoisomerase IIα, 1∶1000 dilution (CalBiochem). Rad21 was visualized in cells expressing myc-tagged Rad21 [49]. When quantifying cellular phenotypes a minimum of 1000 cells were counted per sample. Photomicrographs were acquired with a Zeiss Axioplan2 microscope, an alpha-Plan Fluar 100×/1.45 n.a. objective, and an AxioCam MRC5 camera with Axiovision software (Zeiss).

### Biochemistry

Whole cell extracts were obtained and Western blots performed as previously described [13] using the following antibodies: anti-hRad21 (Abcam, 1∶1500), anti-hShugoshin (Adrian Salic and Tim Mitchison, Harvard Medical School, 1∶1000), anti-CyclinB1 (Abcam, 1∶1500), anti-phospho-H3 (Upstate, 1∶4000), anti-alpha-tubulin (Covance, 1∶1500), anti-Apc2 (Hongtao Yu, 1∶1000) and anti-Smc3 (Bethyl Laboratories, 1∶5000).

### Live cell imaging

HeLa cells were plated onto poly-d-lysine coated 35 mm tissue culture dishes fitted with glass cover-slips (MatTek Cultureware). siRNA transfection and thymidine synchrony was performed as described in Fig. 1 except that upon release from the second thymidine arrest, the standard medium containing the thymidine was exchanged for DMEM without phenol red, supplemented with 10% FBS, penicillin/streptomycin and 200 µM Trolox (Calbiochem). The dishes were transferred to a microscope humidified stage incubator containing 5% CO_2_ at 37°C, 4 hours after release from the early S-phase block. Cells were filmed at 120 second intervals with three z-sections for 16–20 hours, using a Zeiss Axiovert 200M microscope fitted with a 40×/1.3 n.a. Plan-Neofluar objective, an Axiocam HRm camera and using Openlab software.

## Supporting Information

Table S1 On average, in the control cells, mitosis lasted 69 minutes (s.d. = 24 minutes; n = 36), whereas the average time spent in mitosis in PIASγ-depleted cells was 6 hours 35 minutes. The duration of metaphase in controls was somewhat variable, being on average 44 minutes (s.d. = 25 minutes; n = 36), but was rarely longer than 60 minutes. Formation of metaphase plates following nuclear envelope breakdown was achieved in most PIASγ-depleted cells (18/26) within a similar time frame to the control-treated cells. These data are consistent with the analysis of chromosome spreads in that they revealed normal metaphase plate formation in cells lacking PIASγ. Once all of the chromosomes were correctly aligned at the plate, however, anaphase was not initiated on schedule in PIASγ-depleted cells. In 4/20 cells, the metaphase period was very similar to the average metaphase length in control cells and other mitotic stages in these cells were also indistinguishable from the controls, indicating that these four cells may not have received PIASγ-specific siRNA. Even including these cells in the analysis, the average time PIASγ-depleted cells spent in metaphase was ∼2.5× that observed in controls (110 minutes; s.d. = 78 minutes). The maximum metaphase length was 4 hours 10 minutes (∼6 times longer than the average metaphase duration in control cells). After a prolonged period in metaphase, in 11/26 PIASγ-depleted cells, individual chromosomes were seen to leave to the plate, usually reaching the spindle poles. Such chromosomes rarely moved back to the metaphase plate and these cells typically remained in this de-congressed metaphase state for at least another 3 hours. Because in the chromosome spreads (Fig. 1 and Fig. S1) we observed very few separated sister chromatids and because we observed de-congressed metaphases with greatly overcondensed chromosomes (Fig. 1H, Fig. S1N,O), we assume that the chromosomes that left the plate had cohered centromeres (i.e. both sisters moved off the plate together). We cannot say whether these excursions are the cause of the prolonged pre-anaphase delay (perhaps by triggering a checkpoint subsequent to the metaphase-state being achieved), or whether the cells are unable to keep all of the chromosomes on the plate during the prolonged pre-anaphase delay. Remarkably, in some PIASγ-depleted cells that first spent far longer in metaphase that control cells, anaphases ensued following the long metaphase-like delays.
(0.25 MB TIF)

Figure S1 Mitotic progression in control-treated and PIASγ-depleted HeLa cells. Cells were depleted of PIASγ by RNA interference followed by cell cycle synchrony as described in Figure 1, then fixed with 75% methanol 25% glacial acetic acid and stained with Giemsa, as previously described (Gimenez-Abian et al. 2005). (A–E) Control-treated cells, (F–P) PIASγ-depleted cells. (A) Upon nuclear envelope breakdown chromosomes display resolved sister chromatids (except at the centromere regions) and have begun the process of congression. (B,C) Side and polar view, respectively, of control metaphase plates - chromosomes are aligned with their sister chromatids (centromeres and arms) cohered at the equatorial plate. (D) Early anaphase - sisters separate and segregate to the spindle poles synchronously. (E) Mid anaphase - sisters are observed migrating to the poles. (F) Resolution of sister chromatids occurs with the normal timing in PIASγ-depleted cells and is evident after nuclear envelope breakdown. (G–I) Chromosomes congress normally to the metaphase plate in most PIASγ-depleted cells. (J,K) Side and polar view, respectively, of metaphase plates in PIASγ-depleted cells - these cells presumably reached metaphase recently, judging by the degree of chromosome condensation. (L–M) Metaphase arrested PIASγ-depleted cells - correlating with longer times spent arrested in metaphase, chromosomes become over-condensed and metaphase plates become slightly more compact. Proper chromosome alignment of most chromosomes is maintained and centromere regions remain cohered. Chromosome arms fail to open. (N,O) De-congressed metaphases - Around 15% of the PIASγ-depleted metaphase cells lost alignment of one or several chromosomes. This was typically seen after prolonged metaphase arrest (the chromosomes are highly overcondensed indicating a prolonged mitotic block). In such cells, chromosomes off the plate regained strong CENP-E staining at their kinetochores (see Figure 3B–F). (P) Onset of anaphase in a PIASγ-depleted cell (frequency is quantified in Fig. 1J). In this cell, sisters separated asynchronously and arm separation was delayed. Such cells were most frequent after cells had been in metaphase for prolonged periods. It is possible that in such cells, PIASγ-depletion was incomplete, or perhaps these cells had spontaneously leaked through the checkpoint arrest.
(10.56 MB TIF)

Figure S2 PIASγ-specific siRNA treatment causes DNA damage and apoptosis in some cells. (A–F, and I) At a low frequency, HeLa cells treated with PIASγ-specific siRNA reached mitosis with chromosomal damage. Categories of DNA damage included (A) recombination (arrows), (B,C) chromatid breaks and gaps (arrows), (D–F) massive chromosomal breakage resulting in pulverized chromosomes (arrows indicate regions where sisters are seen to be cohered). (I) HeLa cells depleted of PIASγ as described in Figure 1 (main text) were released from early S phase and mitotic cells possessing chromosomal damage were quantified. (G,H and J) The frequency of apoptotic cells was higher after PIASγ-specific siRNA than control treated cells. (K) The number of cells that entered mitosis was reduced after PIASγ-specific siRNA treatment compared with control cells (also see Fig. 1K). To ask if chromosomal damage had arrested some PIASγ-depleted cells in interphase (G2) we added caffeine (an inhibitor of ATM/ATR checkpoint kinases; 2 mM) as well as nocodazole to cells released from early S phase synchrony after PIASγ-depletion. The nocodazole was added upon release and caffeine was added to samples in parallel after 14 hours. Caffeine treatment did not force cells treated with PIASγ-specific siRNA into mitosis.
(8.53 MB TIF)

Figure S3 Statistical analysis of mitotic progression in control and PIASγ-depleted cells. Bar chart showing time spent in prometaphase and metaphase in control HeLa cells and HeLa cells depleted of PIASγ. This analysis corresponds to the live cell analysis described in Figure 2 of the manuscript and Movies 1–4. Each horizontal bar represents a single cell analyzed in the video time-lapse studies. Asterisks (*) indicate cells in which the metaphase plate rotated in the cell so that our observation was from the polar aspect. Thereafter, in these cases we could not determine whether or not chromosomes left the plate. Number Sign (#) indicates cells in which we did not observe metaphase plate formation. In these cases, it is likely that the cells remained in prometaphase, but we cannot rule out the possibility that metaphase plates formed but that the plates remained positioned such that plate formation could not be observed. The grey region indicates cells that ultimately initiated anaphase. Table 1 summarizes the data obtained.
(1.56 MB TIF)

Movie S1 16 hour time-lapse movie of two control HeLa cells expressing H2B-GFP and entering mitosis after the release from a double thymidine block. Selected frames are shown in Fig. 2A.
(8.08 MB MOV)

Movie S2 20 hour time-lapse movie from a wide field of control HeLa cells expressing H2B-GFP and entering mitosis after the release from a double thymidine block. Statistical analysis is shown in Figure S3 and Table S1. Selected frames from selected cells are shown in Fig. 2B.
(12.38 MB MOV)

Movie S3 16 hour time-lapse movie of two PIASγ-depleted HeLa cells expressing H2B-GFP and entering mitosis after the release from a double thymidine block. Both cells congressed their chromosomes with normal timing and then delay in metaphase for 3 hours 26 minutes and 4 hours 18 minutes before initiating anaphase. The cell at the bottom had some laggard chromosomes that finally integrated into the lower pole. Selected frames are shown in Fig. 2C.
(5.39 MB MOV)

Movie S4 20 hour time-lapse movie from a wide field of PIASγ-depleted HeLa cells expressing H2B-GFP and entering mitosis after the release from a double thymidine block. Statistical analysis is shown in Figure S3 and Table S1. Selected frames from selected cells are shown in Fig. 2B.
(14.02 MB MOV)
