# Supplementary figures and images for: PIASγ Is Required for Faithful Chromosome Segregation in Human Cells
Source: PLoS One. 2006 Dec 20;1(1):e53. doi: 10.1371/journal.pone.0000053 (PMC1762334; doi:10.1371/journal.pone.0000053)

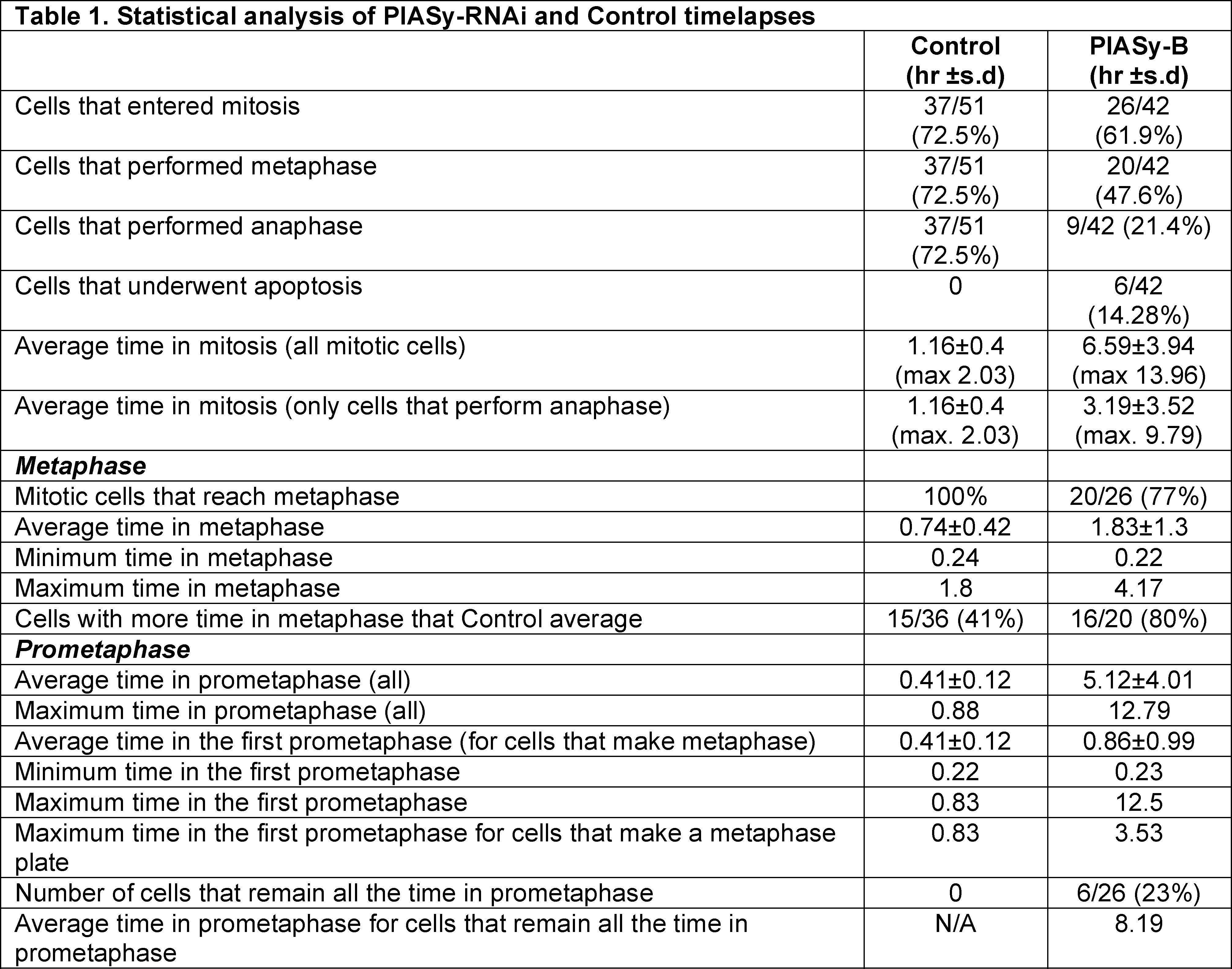

Supplement: Table S1 — On average, in the control cells, mitosis lasted 69 minutes (s.d. = 24 minutes; n = 36), whereas the average time spent in mitosis in PIASγ-depleted cells was 6 hours 35 minutes. The duration of metaphase in controls was somewhat variable, being on average 44 minutes (s.d. = 25 minutes; n = 36), but was rarely longer than 60 minutes. Formation of metaphase plates following nuclear envelope breakdown was achieved in most PIASγ-depleted cells (18/26) within a similar time frame to the control-treated cells. These data are consistent with the analysis of chromosome spreads in that they revealed normal metaphase plate formation in cells lacking PIASγ. Once all of the chromosomes were correctly aligned at the plate, however, anaphase was not initiated on schedule in PIASγ-depleted cells. In 4/20 cells, the metaphase period was very similar to the average metaphase length in control cells and other mitotic stages in these cells were also indistinguishable from the controls, indicating that these four cells may not have received PIASγ-specific siRNA. Even including these cells in the analysis, the average time PIASγ-depleted cells spent in metaphase was ∼2.5× that observed in controls (110 minutes; s.d. = 78 minutes). The maximum metaphase length was 4 hours 10 minutes (∼6 times longer than the average metaphase duration in control cells). After a prolonged period in metaphase, in 11/26 PIASγ-depleted cells, individual chromosomes were seen to leave to the plate, usually reaching the spindle poles. Such chromosomes rarely moved back to the metaphase plate and these cells typically remained in this de-congressed metaphase state for at least another 3 hours. Because in the chromosome spreads (Fig. 1 and Fig. S1) we observed very few separated sister chromatids and because we observed de-congressed metaphases with greatly overcondensed chromosomes (Fig. 1H, Fig. S1N,O), we assume that the chromosomes that left the plate had cohered centromeres (i.e. both sisters moved off the [file pone.0000053.s001.tif]

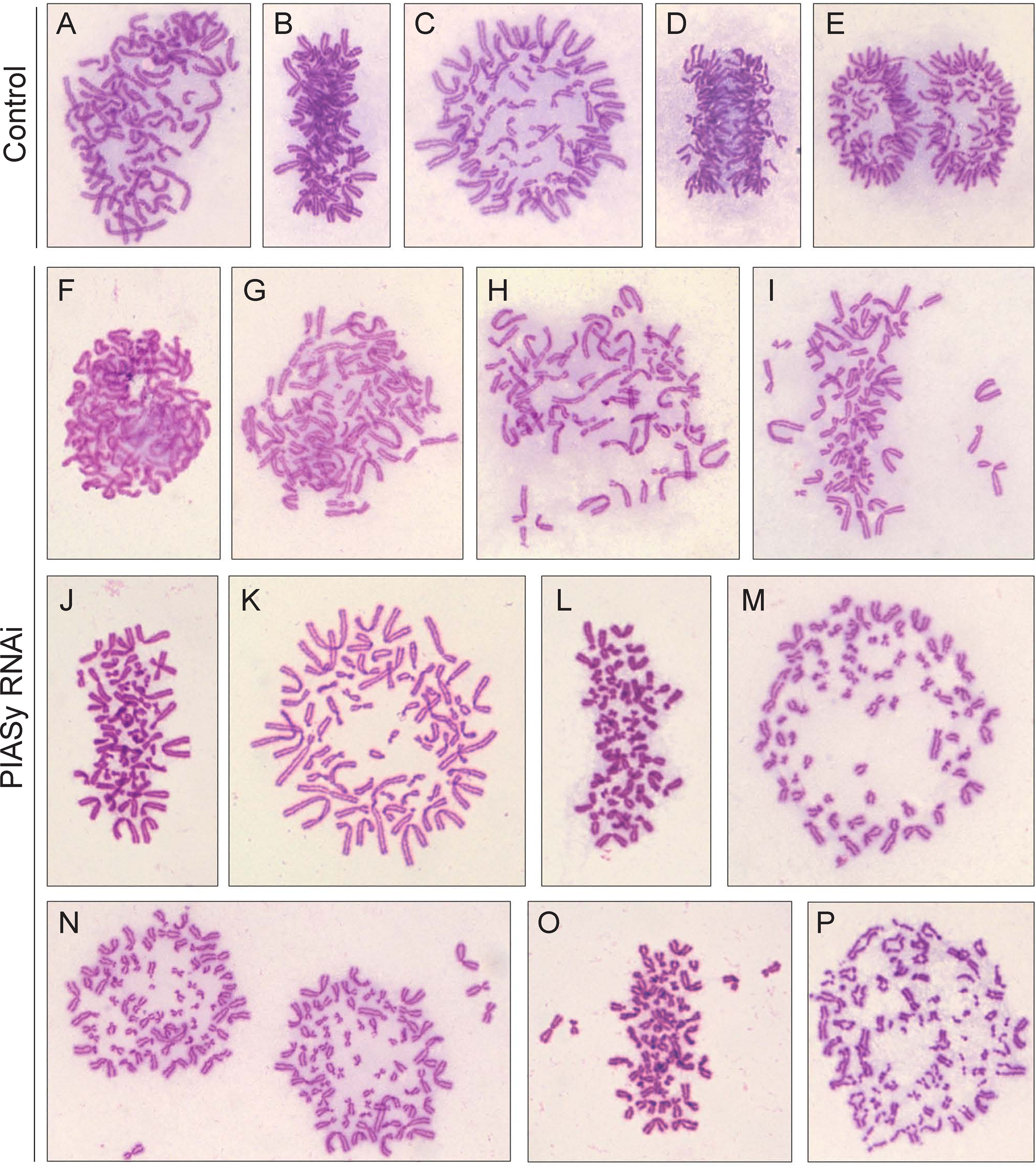

Supplement: Figure S1 — Mitotic progression in control-treated and PIASγ-depleted HeLa cells. Cells were depleted of PIASγ by RNA interference followed by cell cycle synchrony as described in Figure 1, then fixed with 75% methanol 25% glacial acetic acid and stained with Giemsa, as previously described (Gimenez-Abian et al. 2005). (A–E) Control-treated cells, (F–P) PIASγ-depleted cells. (A) Upon nuclear envelope breakdown chromosomes display resolved sister chromatids (except at the centromere regions) and have begun the process of congression. (B,C) Side and polar view, respectively, of control metaphase plates - chromosomes are aligned with their sister chromatids (centromeres and arms) cohered at the equatorial plate. (D) Early anaphase - sisters separate and segregate to the spindle poles synchronously. (E) Mid anaphase - sisters are observed migrating to the poles. (F) Resolution of sister chromatids occurs with the normal timing in PIASγ-depleted cells and is evident after nuclear envelope breakdown. (G–I) Chromosomes congress normally to the metaphase plate in most PIASγ-depleted cells. (J,K) Side and polar view, respectively, of metaphase plates in PIASγ-depleted cells - these cells presumably reached metaphase recently, judging by the degree of chromosome condensation. (L–M) Metaphase arrested PIASγ-depleted cells - correlating with longer times spent arrested in metaphase, chromosomes become over-condensed and metaphase plates become slightly more compact. Proper chromosome alignment of most chromosomes is maintained and centromere regions remain cohered. Chromosome arms fail to open. (N,O) De-congressed metaphases - Around 15% of the PIASγ-depleted metaphase cells lost alignment of one or several chromosomes. This was typically seen after prolonged metaphase arrest (the chromosomes are highly overcondensed indicating a prolonged mitotic block). In such cells, chromosomes off the plate regained strong CENP-E staining at their kinetochores (see Figure 3B–F). (P) Onset of anaphase [file pone.0000053.s002.tif]

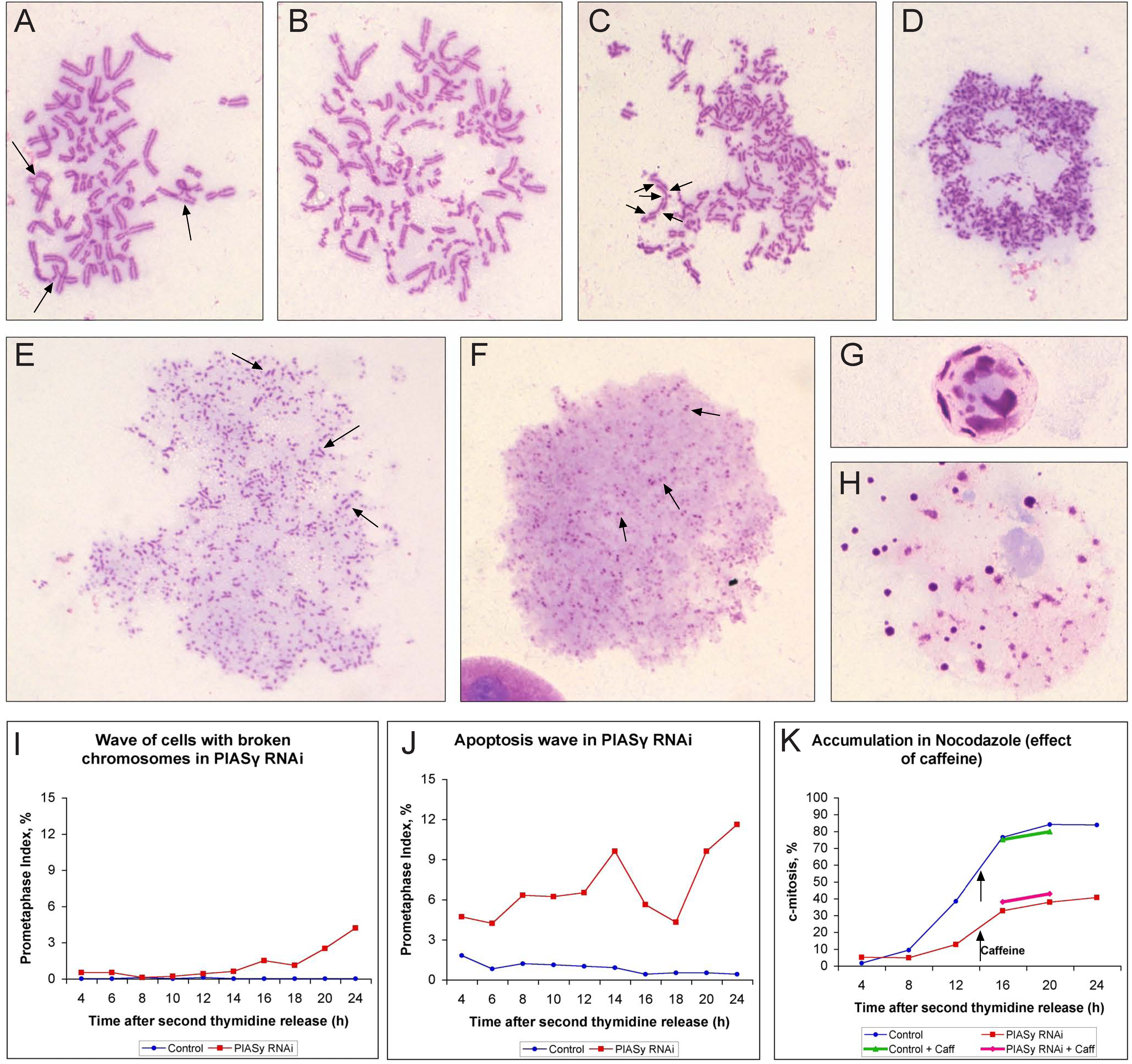

Supplement: Figure S2 — PIASγ-specific siRNA treatment causes DNA damage and apoptosis in some cells. (A–F, and I) At a low frequency, HeLa cells treated with PIASγ-specific siRNA reached mitosis with chromosomal damage. Categories of DNA damage included (A) recombination (arrows), (B,C) chromatid breaks and gaps (arrows), (D–F) massive chromosomal breakage resulting in pulverized chromosomes (arrows indicate regions where sisters are seen to be cohered). (I) HeLa cells depleted of PIASγ as described in Figure 1 (main text) were released from early S phase and mitotic cells possessing chromosomal damage were quantified. (G,H and J) The frequency of apoptotic cells was higher after PIASγ-specific siRNA than control treated cells. (K) The number of cells that entered mitosis was reduced after PIASγ-specific siRNA treatment compared with control cells (also see Fig. 1K). To ask if chromosomal damage had arrested some PIASγ-depleted cells in interphase (G2) we added caffeine (an inhibitor of ATM/ATR checkpoint kinases; 2 mM) as well as nocodazole to cells released from early S phase synchrony after PIASγ-depletion. The nocodazole was added upon release and caffeine was added to samples in parallel after 14 hours. Caffeine treatment did not force cells treated with PIASγ-specific siRNA into mitosis. (8.53 MB TIF) [file pone.0000053.s003.tif]

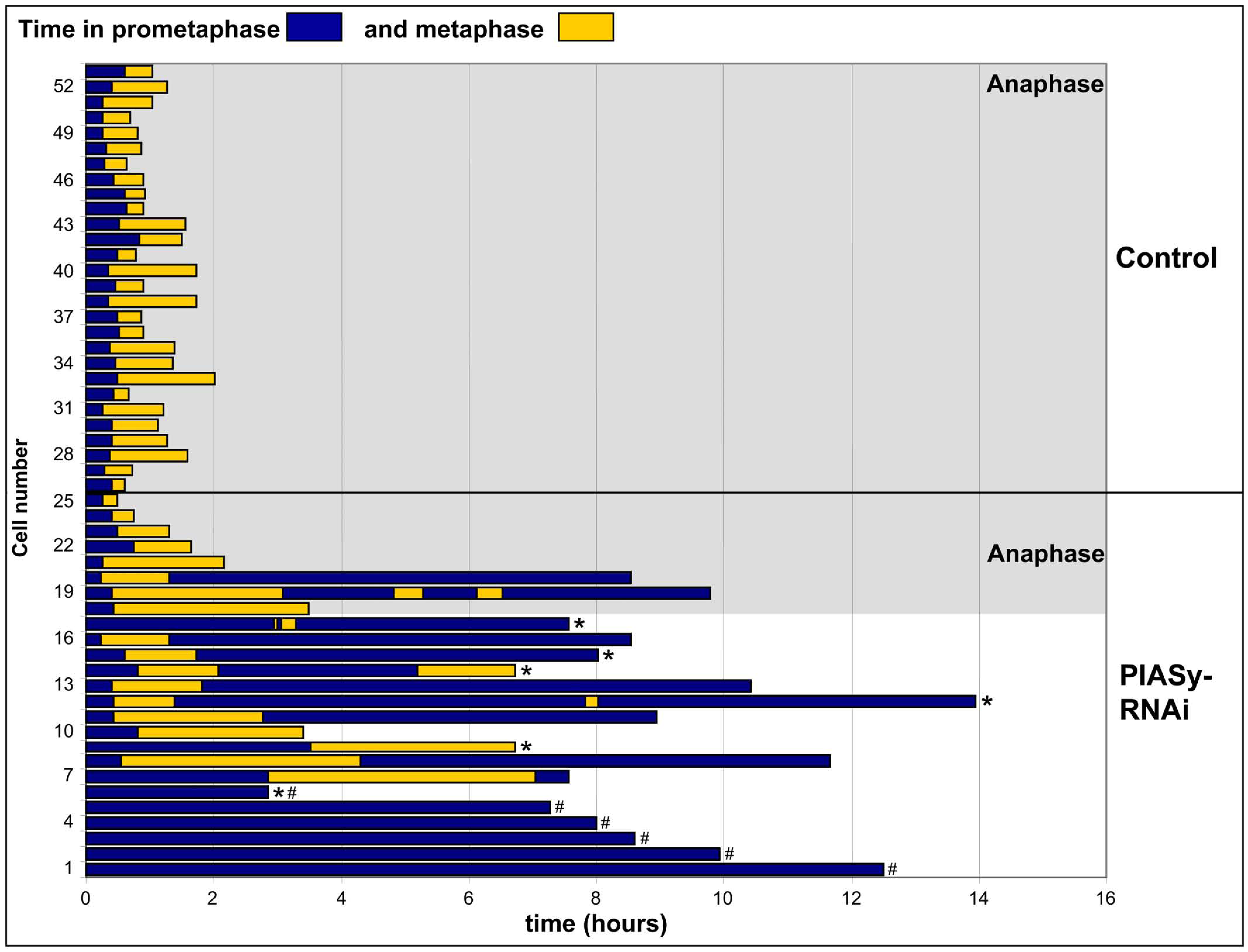

Supplement: Figure S3 — Statistical analysis of mitotic progression in control and PIASγ-depleted cells. Bar chart showing time spent in prometaphase and metaphase in control HeLa cells and HeLa cells depleted of PIASγ. This analysis corresponds to the live cell analysis described in Figure 2 of the manuscript and Movies 1–4. Each horizontal bar represents a single cell analyzed in the video time-lapse studies. Asterisks (*) indicate cells in which the metaphase plate rotated in the cell so that our observation was from the polar aspect. Thereafter, in these cases we could not determine whether or not chromosomes left the plate. Number Sign (#) indicates cells in which we did not observe metaphase plate formation. In these cases, it is likely that the cells remained in prometaphase, but we cannot rule out the possibility that metaphase plates formed but that the plates remained positioned such that plate formation could not be observed. The grey region indicates cells that ultimately initiated anaphase. Table 1 summarizes the data obtained. (1.56 MB TIF) [file pone.0000053.s004.tif]
